# Hepatic fat and dietary intake as determinants of metabolic health in obese adolescents: a cross-sectional MRI study

**DOI:** 10.3389/fnut.2025.1559271

**Published:** 2025-07-09

**Authors:** Hadar Moran-Lev, Ron Sternfeld, Rotem Lazmi, Rivka Ohayon, Rivka Dudi, Avivit Brener, Shira Zelber Sagi, Shlomi Cohen, Ronit Lubetzky, Yftach Gepner

**Affiliations:** ^1^Departments of Pediatric Gastroenterology and Pediatrics, Dana Dwek Children’s Hospital, Tel Aviv Medical Center, Affiliated with the Faculty of Medicine, Tel Aviv University, Tel Aviv, Israel; ^2^Department of Epidemiology and Preventive Medicine, School of Public Health, Faculty of Medicine, Sylvan Adams Sports Institute, Tel-Aviv, Israel; ^3^Departments of Pediatric Endocrinology, Dana Dwek Children’s Hospital, Tel Aviv Medical Center, Affiliated with the Faculty of Medicine, Tel Aviv University, Tel Aviv, Israel; ^4^School of Public Health, University of Haifa, Haifa, Israel

**Keywords:** metabolic health, obesity, adolescents, hepatic fat content, diet

## Abstract

**Background:**

Obesity in youth is highly associated with metabolic risk. However, a subset of individuals maintains metabolic health despite the presence of obesity. This study aims to identify key factors associated with a metabolically healthy obese (MHO) in adolescents.

**Methods:**

This cross-sectional study included 31 adolescents with obesity [median age: 14 y, median body mass index (BMI) Z-score: 2.58] categorized as MHO or metabolically unhealthy obese (MUO) based on the presence of one or more metabolic syndrome criteria. A comprehensive evaluation included MRI assessments of abdominal adipose tissue distribution and hepatic fat content (HFC), physiological and metabolic assessment, serum biomarkers, prenatal and sociodemographic characteristics, and dietary habits.

**Results:**

Compared to the MUO, MHO individuals exhibited significantly lower HFC (*p* = 0.01), liver enzymes (*p* = 0.004), and experienced a lower risk of complications during birth (*p* = 0.03). Additionally, MHO participants had significantly lower intake of total calories (*p* = 0.04), animal protein (*p* = 0.005), red meat (*p* = 0.02), sodium (*p* = 0.027), palmitic acid (*p* = 0.04), stearic acid (*p* = 0.029), arachidonic acid (*p* = 0.005) and calories from ultra-processed grains (*p* = 0.049) compared to their MUO counterparts.

**Conclusion:**

Adolescents with MHO show lower hepatic fat, improved liver markers, and healthier dietary patterns than MUO peers. These findings underscore the potential influence of prenatal and lifestyle factors in distinguishing metabolic health profiles in adolescents with obesity.

**Clinical trial registration:**

https://clinicaltrails.gov/study/NCT06032312; NCT06032312.

## Introduction

The global epidemic of childhood obesity is characterized by a rising prevalence of endocrine, metabolic, and cardiovascular comorbidities and represents one of the major public health challenges of our time (1–4). Recent data underscore alarming trends in childhood obesity and its associated metabolic syndrome (MS) features (2, 4–6). MS refers to a cluster of cardio-metabolic risk factors, including high blood pressure, high blood sugar, excess abdominal adiposity, and abnormal lipid profiles (7). The presence of multiple risk factors extends the likelihood of developing type 2 diabetes mellitus (T2DM) and cardiovascular diseases (8). The increasing prevalence of childhood obesity has raised concerns about reduced life expectancy, as early-onset obesity significantly elevates the risk of chronic conditions such as cardiovascular disease and type 2 diabetes, which can lead to premature mortality (9, 10).

A unique subgroup of adolescents with obesity, termed ‘metabolically healthy obese’ (MHO), has drawn attention due to its atypical profile, lacking the metabolic risk factors typically associated with obesity (11–14). Despite their obesity status, youngsters with MHO exhibit a favorable metabolic profile, characterized by preserved insulin sensitivity, and normal blood pressure, glucose regulation, lipid and liver enzyme levels, as well as balanced hormonal, inflammatory, and immune profiles (7, 15–18). Several prospective studies in both adolescents and adults have demonstrated that MHO is not consistently linked to increased cardiovascular risk or all-cause mortality (19–21). Therefore, identifying and understanding this MHO subgroup among adolescents with obesity is crucial for uncovering mechanisms that may prevent the development of clustered cardiometabolic risk factors and related diseases, enabling more informed clinical, preventive, and therapeutic decisions (4, 11, 18, 22, 23).

Several studies have examined the predictors and risk factors associated with MUO individuals (22, 23). However, these studies focused primarily on adult population and have yielded mixed results, due to variations in the definitions of MHO and criteria used for its classification.

Moreover, previous studies have not comprehensively investigated the interaction among perinatal history, sociodemographic factors, body composition, physical activity, and dietary composition in the pediatric population with obesity, nor have they attempted to establish relationships between these variables. Therefore, the primary objective of this study was to assess factors associated with MHO within a framework encompassing sociodemographic, clinical, body composition, nutritional, and physical performance aspects among adolescents with obesity. Given the emerging relevance of Hepatic fat content (HFC) in metabolic health, we hypothesize that variations in hepatic fat levels will contribute to the process of distinguishing between metabolically healthy and unhealthy states in the context of childhood obesity.

## Methods

### Study design

This prospective observational study was performed in the Nutrition and Obesity Clinic of the Pediatric Gastroenterology Institute, “Dana Dwek” Children’s Hospital of the Tel Aviv Soursky Medical Center, which is a tertiary care pediatric hospital. Participants under routine care at the Clinic were invited to participate in the study after obtaining parental consent. The study protocol included a clinic visit to obtain physical examination, anthropometric measurements and record prenatal and sociodemographic information. Participants then completed a full day of assessments, which included magnetic resonance imaging (MRI) and magnetic resonance spectroscopy (MRS) scans, along with various physiological performance tests, such as isometric mid-thigh pull and handgrip dynamometer strength tests and resting metabolic rate (RMR) measurements (see below for details). Additionally, self-reported data on physical activity levels and dietary habits were collected. The study was approved by the Institutional Review Board of Tel Aviv Medical Center (TLV-0799-20). All parents provided written informed consent prior to the participant’s enrollment (clinical trial registration number: NCT06032312).

### Study population

Eligible participants for the study were adolescents with obesity, treated at the Obesity Clinic between January 2021 and August 2023. Exclusion criteria included any underlying disease or treatments potentially affecting body composition or metabolic risk (e.g., anti-inflammatory medications, Metformin), or inability to complete an MRI scan.

Initially, 52 adolescents were eligible for the study. Of these, nine were excluded due to underlying conditions or medication use (chronic kidney disease, celiac disease, neurologic disability, or regular use of metformin or risperidone), and twelve declined to participate. The final sample included 31 adolescents: 15 in the MUO group and 16 in the MHO group.

### Demographic and clinical variables

Information retrieved from the medical files of the study participants included:

1. Sociodemographic characteristics: age, sex, home address.
2. Medical history: complication during pregnancy, perinatal characteristics (birth weight, gestational age, mode of delivery), medications, and family history of cardiometabolic diseases (diabetes, hypertension, dyslipidemia, cardiovascular disease, and cerebrovascular episodes) among first- and second-degree relatives.
3. Physical examination: systolic and diastolic blood pressure and anthropometric measurements (height and weight conducted following standardized protocols). BMI, height and birth weight Z-scores were calculated by means of sex and age-specific BMI reference values from the Center for Disease Control and Prevention growth charts (24).
4. Documented blood markers at study entry included: total cholesterol, low-density lipoprotein cholesterol (LDL-c), high-density lipoprotein-cholesterol (HDL-c), triglycerides (TG), alanine transaminase (ALT), fasting plasma glucose (FPG), and insulin.
5. Socioeconomic position (SEP) was determined by the patient’s home address according to the Israel Central Bureau of Statistics’ Characterization and Classification of Statistical Areas within Municipalities and Local Councils by the Socio-Economic Level of the Population 2015 (25).
6. The quality of life (QoL) of the study participants was assessed with the Pediatric Quality of Life Inventory (PedsQL), version 3.0 (26). PedsQL is a 23-item assessment of physical, emotional, social, and school functioning.

### RMR assessment

RMR was measured in a metabolic unit using an indirect calorimeter device (Quark RMR; Cosmed, Rome, Italy). Indirect calorimetry was based upon the ratio of gas exchange, specifically, carbon dioxide production (VCO_2_) versus oxygen consumption (VO_2_), which reflects energy metabolism according to the Weir equation (27). The 20-min RMR measurement was performed in a supine position after 8 h of fasting and a minimum of 24 h of abstaining from alcohol, caffeine, smoking, and exercise. Gas and turbine calibrations were performed before each test. The first 4 min, also known as the adaptation phase, were excluded from the mean RMR, resulting in the utilization of only the final 16 min to determine RMR values.

### Nutritional assessment

A registered dietitian administered a self-reported semi-quantitative food frequency questionnaire (FFQ) to assess the participant’s nutritional intake. The 126-item questionnaire aimed to estimate the participant’s dietary habits over the previous year (28). In addition to the measurement of energy intake, the FFQ data underwent a further analysis to generate values for food group consumption, including a macro- and micronutrient breakdown. Ultra-processed foods were classified as falling under NOVA group 4 according to the NOVA food classification system (29). Group 4 includes industrial formulations typically containing five or more ingredients, such as sugars, oils, fats, salt, preservatives, colorings, flavorings, and other additives designed to enhance shelf life, taste, and texture. Examples of ultra-processed foods include sugary breakfast cereals, packaged snacks, and soft drinks, which undergo extensive processing and contain minimal whole food components. This standardized classification was applied to all dietary data to ensure reproducibility and consistency in defining ultra-processed food intake.

The Mediterranean diet score, developed by Panagiotakos et al., evaluates adherence to the Mediterranean diet, with scores ranging from 0 to 55 (30). It assesses the frequency of consumption of various food groups. Including non-refined cereals, fruits, vegetables, legumes, potatoes, fish, meat, poultry, full-fat dairy products, olive oil, and alcohol. Each item within these groups receives a score from 0 to 5 based upon monthly consumption frequency (from never to >18 servings per month). These analyses utilized the Israeli national nutrient database from the Israeli Ministry of Health (‘Tzameret’), as per the Food and Nutrition Services and Public Health Services guidelines [2008] in the Israeli Nutrient Database (31).

### Body composition and fat distribution assessment

1. Fat mass, fat-free mass, and skeletal muscle mass were determined by means of a multi-frequency SECA body composition analyzer (mBCA 514 Medical, Hamburg, Germany), utilizing bioelectrical impedance analysis. The calculated bioelectrical impedance analysis variables included appendicular skeletal muscle mass (i.e., skeletal muscle mass of all four limbs) and muscle-to-fat ratio (i.e., appendicular skeletal muscle mass [kg]/total fat mass [kg]). Participants wore light clothing during these assessments and removed all jewelry, socks, and shoes. These measurements were conducted in the morning following an overnight fast.
2. Fatty liver was determined by clinical radiologists via abdominal ultrasonography by a Logic Q700 MR machine (GE, Milwaukee, WI, USA) and a 3.5 MHz probe. The ultrasound images included: (i) sagittal view of the right lobe of the liver and right kidney, (ii) transverse view of the left lateral segment of the liver and spleen, and (iii) transverse view of the liver to assess for altered echo texture. Fatty liver infiltration was identified by an increase in liver echogenicity compared to the echogenicity of the renal cortex, with the diaphragm and intrahepatic vessels appearing normal.
3. Abdominal adipose tissue distribution and HFC were evaluated with MRI and MRS, respectively, by means of the Siemens MAGNETOM Prisma 3-Tesla MRI scanner at the Alfredo Federico Strauss Center at Tel Aviv University. MRI scans were conducted at three axial levels (L5-L4, L4-L3, and L3-L2) to precisely determine visceral fat distribution. The mean values for visceral adipose tissue, deep subcutaneous adipose tissue, and superficial subcutaneous adipose tissue were calculated based upon the three axial slices.

HFC was determined by means of MRS, a well-validated analytical technique that detects radiofrequency electromagnetic signals emitted by atomic nuclei within molecules (32, 33). The total hepatic fat fraction within the image was determined as the ratio of the sum of the area under all fat peaks to the sum of the area under all fat and water peaks.

### Strength performance and physical activity assessment

Muscle strength and function were assessed with an isometric mid-thigh pull and handgrip dynamometer (34). A handgrip strength test was employed to measure upper limb strength by means of a handgrip dynamometer (Baseline® 200 lb. Capacity, New Jersey, USA) (35). Finally, the participants were instructed to wear an accelerometer (GT9X©; ActiGraph, Pensacola, FL, USA) for seven consecutive days to track their sedentary and vigorous activity periods. Data extraction from the device included calibrating the raw data and identifying wear/non-wear episodes to gather validated information on the physical activity levels. Physical activity intensity was classified using established count thresholds: sedentary time as <100 counts per minute (≤1.5 METs) and vigorous activity as ≥5,999 counts per minute (≥6 METs). This standardized approach for quantifying sedentary and vigorous activity follows Troiano et al. (35).

### Classification of metabolically unhealthy obese and definition of metabolic conditions

The diagnose of MUO was based on the consensus-based definition and includes all children and adolescence with a BMI over the 95th percentile, along with one or more of the following criteria: HDL-c < 40 mg/dL (or <1.03 mmol/L), TG ≥ 150 mg/dL (or ≥1.7 mmol/L), systolic and diastolic blood pressure ≥90th percentile, and FPG ≥ 100 mg/dL (or ≥5.5 mmoL/L) (7).

Insulin resistance was determined with the Homeostatic Model Assessment of Insulin Resistance (HOMA-IR) formula: fasting insulin (μU/mL) × fasting glucose (mmol/L)/22.5 (36).

A diagnosis of MASLD was established according to the recent multisociety statement as any case of hepatic steatosis in the setting of cardiometabolic risk factors (37).

### Outcomes

#### Primary outcome

The primary outcome of this study was HFC, measured using magnetic resonance spectroscopy (MRS), as a key differentiator between MHO and MUO adolescents.

#### Secondary outcomes

1. Assessment of lifestyle factors, such as physical activity levels and dietary quality as predictors of metabolic health in adolescents with obesity.
2. Examination of sociodemographic and perinatal factors, including birth history and socioeconomic position, that may influence metabolic health.
3. Evaluation of muscle strength and RMR to explore their associations with metabolic health among adolescents with obesity.

### Statistical analysis

A convenient sample of 30 participants was chosen after determining the sample size for assessing trends for metabolic health among adolescents with obesity. The power analysis was conducted based on HFC content, our primary outcome, based on a previous study (38) that assessed the metabolic function of HFC among 31 adults. HFC was 5-fold (3.6% ± 0.5 vs., 15.3% ± 3.5) in the MUO group, yielding an effect size of 7.62 (Cohen’s d). With *α* = 0.05, this sample size provides over 99% power, confirming adequacy for detecting significant differences in HFC between groups.

Adolescents were matched for age and BMI Z score to minimize confounding variables and enhance result comparability. Epidemiological data and participant descriptors measured on continuous scales were reported as means and standard deviations, while categorical variables were presented as percentages of the number of participants. Parametric tests, including the Student’s t-test for continuous variables, were employed for normally distributed data, while non-parametric tests, such as the Mann–Whitney U test, were utilized for non-normally distributed continuous variables. Levene’s test assessed the equality of group variances for continuous variables. The Chi-squared test was applied for categorical variable analysis. Logistic regression analysis was employed to assess the impact of HFC on metabolic health, adjusting for BMI, sex, age, and total calories. Additionally, linear regression using the enter method was applied to evaluate the independent effect of HFC on FPG, a primary metabolic health criterion, while adjusting for saturated fatty acid (SFA) and animal protein. Statistical significance was set at *p* < 0.05 following Bonferroni correction for multiple comparisons. Due to the relatively small sample size and the number of outcomes examined, this conservative method was selected to limit type I error and avoid misleading inferences. Data values were excluded if they deviated more than three times the standard deviation from the mean in order to ensure that extreme values do not unjustifiably influence the statistical results. The statistical analyses were carried out using SPSS V. 25 (SPSS Inc., Chicago, IL, USA) software, and Prism V. 8 (GraphPad, La Jolla, CA, USA).

## Results

### Clinical factors and sociodemographic data

The sociodemographic and clinical data of all participants are detailed in Table 1. There were 10 females and 6 males in the MHO group and 7 females and 8 males in the MUO group. The mean age and the BMI were similar for both groups. As anticipated, the MHO group had significantly higher levels of HDL-c and TG concentration as well as higher percentage of hypertension and insulin resistance (Table 1). Moreover, the MUO group also had a significantly higher elevation in ALT compared to the MHO group (45.0 ± 23.6 U/L vs. 21.3 ± 8.1 U/L. *p* = 0.004). This latter finding was consistent with the substantially higher incidence of fatty liver observed on the ultrasound scans in the MUO group (MHO: 15.3% vs. MUO: 76.9%; *p* = 0.002).

**Table 1:** Clinical and sociodemographic characteristics of the study population.

Variables	Metabolically healthy obese (*n* = 16)	Metabolically unhealthy obese (*n* = 15)	*p*-value
Age (y)	14.5 ± 1.9	14.2 ± 2.0	0.64
Female, *n* (%)	10 (62.5)	7 (46.7)	0.37
Weight (kg)	100.0 ± 17.1	108.4 ± 12.1	0.28
Height (m)	1.64 ± 0.1	1.67 ± 0.1	0.41
Height Z-score	0.35 ± 0.93	0.69 ± 0.98	0.33
BMI (kg/m^2^)	37.1 ± 6.0	38.7 ± 8.3	0.54
BMI Z-score	2.47 ± 0.51	2.77 ± 0.67	0.18
Ultrasonography fatty liver, *n* (%)	2 (15.3)	10 (76.9)	**0.002**
Hypertension, *n* (%)	0 (0.0)	4 (26.6)	**0.02**
Blood tests			
Total Cholesterol (mg/dL)	150.9 ± 20.2	165.4 ± 28.3	0.13
LDL-c (mg/dL)	83.0 ± 18.3	96.4 ± 25.7	0.13
HDL-c (mg/dL)	44.7 ± 5.7	37.0 ± 5.5	**0.001**
TG (mg/dL)	96.5 ± 27.0	152.2 ± 66.7	**0.01**
ALT (u/L)	21.3 ± 8.1	45.0 ± 23.6	**0.004**
FPG (mg/dL)	89.8 ± 6.5	99.0 ± 20.6	0.11
Insulin (μU/mL)	22.0 ± 10.7	48.9 ± 23.4	**0.009**
Homa-IR (units)	4.1 ± 2.8	13.2 ± 10.2	**0.02**
Prenatal characteristics			
Birth weight (kg)	3.2 ± 0.6	3.0 ± 0.7	0.54
Birth week (weeks)	38.0 ± 1.5	38.5 ± 1.4	0.43
Birth weight-for-birth week Z-score	0.12 ± 1.35	-0.53 ± 1.70	0.24
High-risk pregnancy, *n* (%)	2 (12.5)	7 (46.7)	**0.03**
Cesarean section, *n* (%)	5 (26.3)	3 (21.4)	0.54
Breastfeeding, *n* (%)	9 (56.2)	6 (42.8)	0.46
Sociodemographic characteristics			
SPS status index	0.74 ± 0.54	0.66 ± 0.59	0.71
Physical functioning score	69.0 ± 14.7	68.7 ± 22.5	0.97
Emotional functioning score	60.0 ± 15.8	75.8 ± 21.6	0.06
Social functioning score	68.6 ± 20.8	78.7 ± 15.9	0.19
School functioning score	46.8 ± 8.7	62.5 ± 13.0	**0.003**
Total QoL score	59.8 ± 11.2	71.4 ± 12.1	**0.03**
Cardiometabolic risk, *n* (%)	9 (56.2)	12 (80.0)	0.15
Family history of bariatric surgery, *n* (%)	3 (18.7)	7 (50.0)	0.07

Values are means ± SD. BMI, body mass index; LDL-c, low-density lipoprotein; HDL-c, high-density lipoprotein; TG, triglycerides; ALT, alanine transaminase; FPG, fasting plasma glucose; Homa-IR, homeostatic model assessment for insulin resistance; SPS, socioeconomic position; QoL, quality of life. Bold text indicates significant findings *p* < 0.05 (*t*-tests for continuous variables; Chi-square for categorical variables).

Significantly more high-risk pregnancies (defined as any pregnancy that required special care, such as intrauterine growth restriction, maternal hypertension, etc.) were reported for the adolescents with MUO (46.7%) compared to the adolescents with MHO (12.5%) (*p* = 0.03), However, no differences were noted in terms of birth weight, weeks’ gestation, or mode of delivery. In addition, MUO adolescents had significantly higher score in school functioning and overall QoL compared to their MHO counterpart (*p* < 0.05).

### Body composition, fat distribution, strength performance and physical activity associated with metabolic health

**Figure 1:**
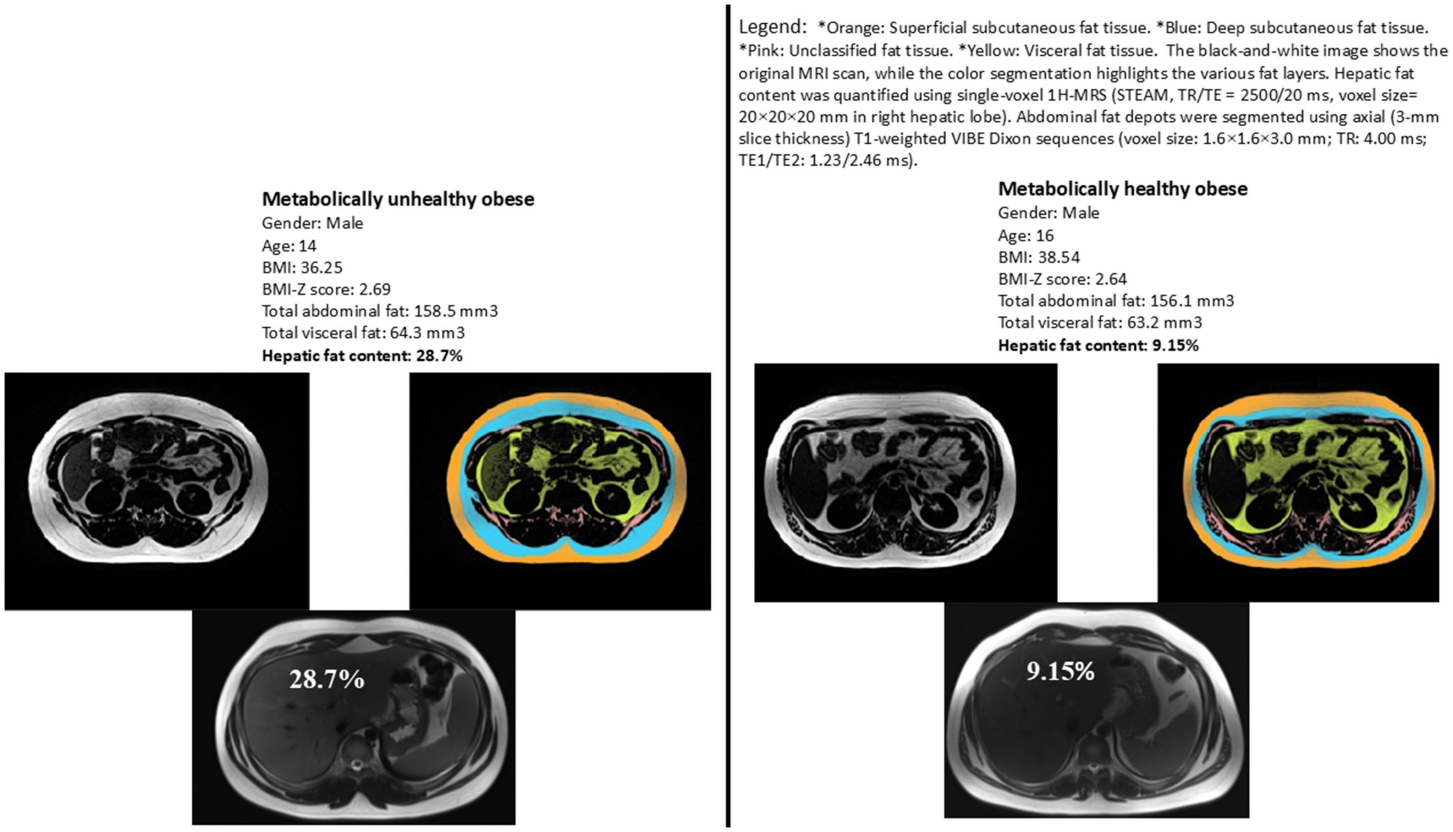
Representative images illustrating hepatic fat content for two matched subjects from each study group. Matching criteria include sex, BMI z-score, total abdominal fat, and visceral fat. Variations in hepatic fat content are evident between the two subjects, highlighting the difference between metabolically healthy and unhealthy obese adolescents.

Physiological and body composition parameters for the two study groups are listed in Table 2. HFC, measured by MRS, was significantly elevated in the MUO group compared to the MHO group (14.0 ± 9.8% vs. 6.1 ± 3.2%, respectively, *p* = 0.01) (Figure 1). However, the levels of fat mass, fat-free mass and muscle-to-fat ratio were comparable for the MHO and MUO groups. The total abdominal fat, visceral adipose tissue, deep subcutaneous adipose tissue and superficial subcutaneous adipose tissue distribution was also similar for the MHO and MUO groups (Table 2). Interestingly, there were no significant differences between the MHO and MUO groups in RMR (1962 ± 427 vs. 2,259 ± 379; *p* = 0.07), nor for the physical activity and strength measures of sedentary time per day (58.2 ± 8.4% vs. 59.1 ± 7.6%; *p* = 0.81), handgrip max strength (23.1 ± 7.7 kg vs. 25.2 ± 7.3 kg, *p* = 0.43), and isometric mid-thigh pull max (169.4 ± 38.9 kg vs. 165.9 ± 32.1 kg, *p* = 0.80) (Table 2).

**Table 2:** Metabolic and physiological parameters in MHO and MUO.

Variables	Metabolically healthy obese (*n* = 16)	Metabolically unhealthy obese (*n* = 15)	*p*-value
Metabolic			
Resting metabolic rate (kcal/day)	1962 ± 427	2259 ± 379	0.07
Respiratory quotient	0.85 ± 0.21	0.75 ± 0.12	0.15
Body composition and fat pools			
Body fat (%)	46.2 ± 5.9	43.9 ± 7.2	0.35
Fat mass (kg)	45.6 ± 10.2	48.7 ± 18.2	0.58
Fat-free mass (kg)	53.1 ± 11.6	59.6 ± 9.8	0.11
Skeletal muscle mass (kg)	25.0 ± 6.3	29.3 ± 5.7	0.06
Appendicular skeletal muscle mass (kg)	18.75 ± 4.75	21.99 ± 4.3	0.06
Muscle-to-fat ratio (kg)	0.42 ± 0.1	0.48 ± 0.1	0.23
HFC (%)	6.1 ± 3.2	14.0 ± 9.8	**0.01**
Total abdominal fat (mm^3^)	179.5 ± 42.3	209.8 ± 72.1	0.20
Visceral adipose tissue (mm^3^)	48.6 ± 9.2	53.4 ± 12.7	0.29
Deep subcutaneous adipose tissue (mm^3^)	50.4 ± 20.0	51.2 ± 12.8	0.91
Superficial subcutaneous adipose tissue (mm^3^)	80.4 ± 28.6	86.5 ± 37.3	0.64
Physical activity and performance			
Days of accelerometer wearing (days)	7.4 ± 0.8	8.0 ± 1.8	0.36
Sedentary time (%/day)	58.2 ± 8.4	59.1 ± 7.6	0.81
Vigorous time (%/day)	0	0	
Hand grip strength max (kg)	23.1 ± 7.7	25.2 ± 7.3	0.43
Isometric mid-thigh pull max (kg)	169.4 ± 38.9	165.9 ± 32.1	0.80

Values are means ± SD. Bold text indicates significant findings *p* < 0.05 (*t*-tests for continuous variables; Chi-square for categorical variables). HFC, hepatic fat content.

### Differences in dietary patterns between metabolically healthy and unhealthy obese adolescents

Table 3 depicts the self-reported dietary consumption patterns of the MHO and the MUO groups.

**Table 3:** Differences in self-reported nutrient intake between healthy and metabolically unhealthy obese adolescents.

Variables	Metabolically healthy obese (*n* = 16)	Metabolically unhealthy obese (*n* = 15)	*p*-value
Total calories (kcal/day)	1655 ± 475	2071 ± 517	**0.04**
Carbohydrate (g/day)	177 ± 67	208 ± 65	0.23
Fat (g/day)	66 ± 19	83 ± 24	0.06
Protein (g/day)	75 ± 18	110 ± 32	**0.003**
Added sugars (g/day)	18 ± 13	21 ± 14	0.66
Fructose (g/day)	18 ± 10	23 ± 10	0.26
Dietary fiber (g/day)	26 ± 15	32.5 ± 11	0.28
Saturated fat (g/day)	20 ± 5	25 ± 9	0.09
Palmitic acid (g/day)	10.5 ± 2.8	13.7 ± 4.9	**0.04**
Stearic acid (g/day)	4.1 ± 1.1	5.5 ± 2.1	**0.029**
Arachidonic acid (g/day)	0.14 ± 0.1	0.30 ± 0.1	**0.002**
Animal protein (g/day)	49 ± 17	76 ± 27	**0.005**
Red meat calories (kcal/day)	40 ± 50	93 ± 59	**0.02**
Calcium (mg/day)	847 ± 352	962 ± 368	0.41
Iron (mg/day)	10.2 ± 4.2	15.0 ± 5.1	**0.01**
Sodium (mg/day)	3050 ± 1090	4084 ± 1056	**0.02**
Ultra-processed food calories (kcal/day)	364 ± 210	467 ± 247	0.26
Ultra-processed grains calories (kcal/day)	67 ± 37	116 ± 78	**0.049**
Mediterranean diet score (units)	29 ± 6	27 ± 5	0.22

Values are means ± SD. Bold text indicates significant findings *p* < 0.05 (*t*-tests for continuous variables; Chi-square for categorical variables).

MUO individuals had a significantly higher total daily calorie consumption (MHO: 1655 ± 475 kcal/day vs. MUO: 2071 ± 517 kcal/day; *p* = 0.04). Furthermore, there was a substantial increase in daily protein consumption by MUO individuals (MHO: 75 ± 18 g/day vs. MUO: 110 ± 32 g/day; *p* = 0.003), along with animal protein (MHO: 49 ± 17 g/day vs. MUO: 76 ± 27 g/day; *p* = 0.005), red meat calories (MHO: 40 ± 50 kcal/day vs. MUO: 93 ± 59 kcal/day; *p* = 0.02), and iron intake (MHO: 10.2 ± 4.2 mg/day vs. MUO: 15.0 ± 5.1 mg/day; *p* = 0.01). Likewise, MUO individuals exhibited significantly higher daily intakes of palmitic acid, stearic acid and arachidonic acid (*p* < 0.05). In addition, adolescents with MUO consumed significantly higher calories from ultra-processed grains (MHO: 67 ± 37 kcal/day vs. MUO: 116 ± 78 kcal/day; *p* = 0.05) and significantly higher sodium (MHO: 3050 ± 1,090 mg/day vs. MUO: 4084 ± 1,056 mg/day; *p* = 0.02).

### The independent effect of HFC on metabolic health

Table 4 displays the odds ratios (OR) for MUO status based upon the HFC while adjusting for various covariates. Model 1 represents the unadjusted OR of 1.21 (95% confidence interval [CI]: 1.02–1.41; *p* = 0.02). Model 2 adjusted for BMI, age and sex revealed an OR of 1.19 (95% CI: 1.00–1.41; *p* = 0.04). Model 3 included all the covariates and incorporated total calorie intake demonstrated borderline statistical significance [OR of 1.20 (95% CI: 0.99–1.46; *p* = 0.06)].

**Table 4:** Odds ratios for unhealthy metabolic obesity according to hepatic fat content percentage, with adjustments for covariates.

Models	OR (95% CI)	*p*-value
Unadjusted	1.21 (1.02–1.41)	**0.02**
Adjusted for BMI, age, and sex	1.19 (1.00–1.41)	**0.04**
Adjusted for BMI, age, sex, and total calories	1.20 (0.99–1.46)	0.06

Multivariable logistic regression analysis assessed the association between hepatic fat content and metabolic health. OR, odds ratio; CI, confidence interval; BMI, body mass index. Bold text indicates significant findings *p* < 0.05.

**Figure 2:**
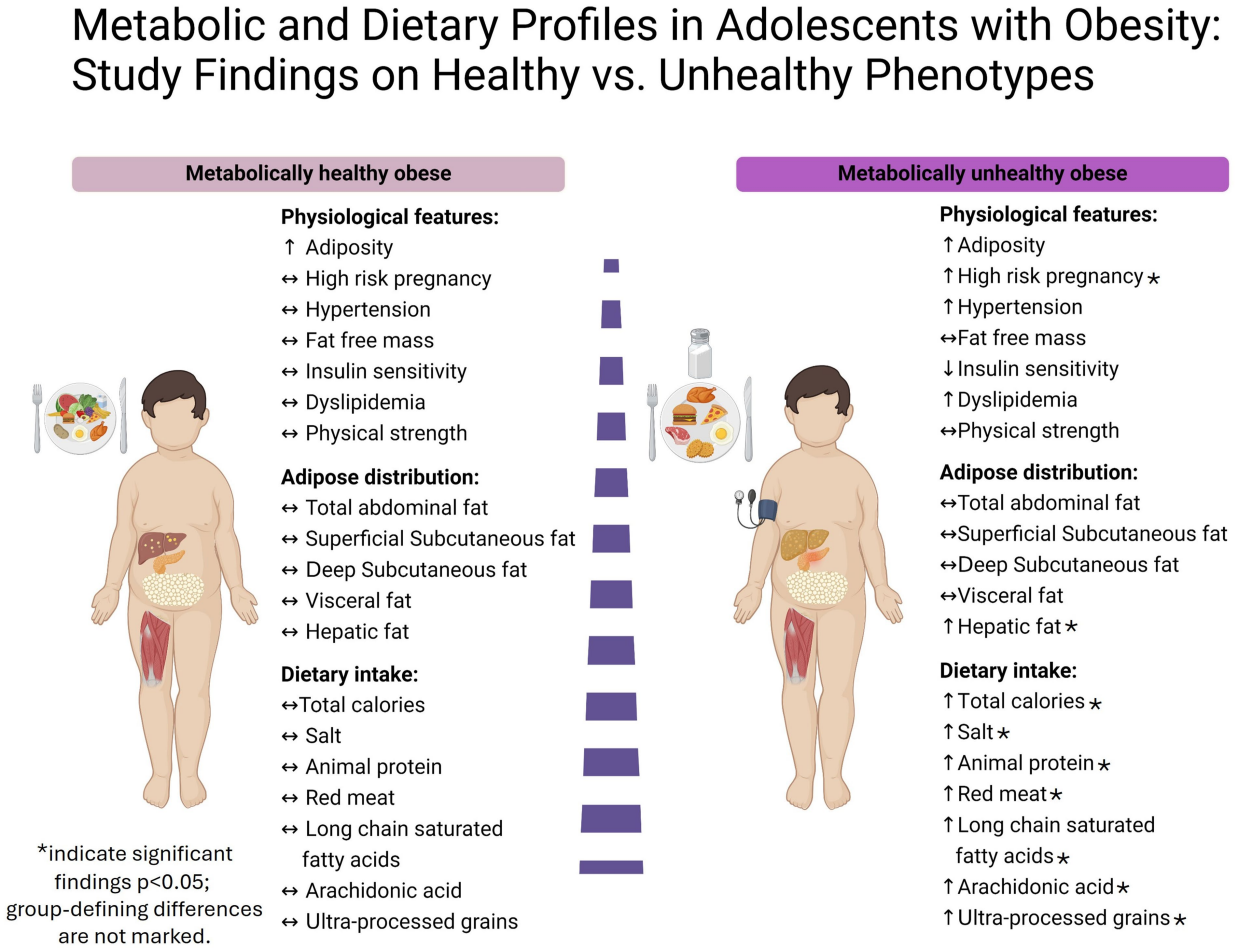
Key findings from the MHO and MUO group comparison.

Figure 2 presents the key findings from the MHO and MUO group comparison. It highlights the elevated hepatic fat content (HFC) in the MUO group, alongside their higher intake of total calories, sodium, animal protein, red meat, long-chain saturated fatty acids, and ultra-processed grains. Additionally, the figure shows a greater prevalence of high-risk pregnancies in the MUO group. Notably, both groups exhibit similar levels of fat-free mass, fat distribution, and physical strength.

## Discussion

In this study, we observed marked differences in fatty liver presence and extent between adolescents with MHO and those with MUO. Additional distinctions emerged in the history of high-risk pregnancies and dietary habits between the groups. Specifically, adolescents with MUO reported greater consumption of calories, animal protein, sodium, arachidonic acid, long-chain saturated fatty acids, and ultra-processed grains compared to their MHO counterparts.

Our findings reveal that a higher proportion of adolescents in the MUO group were born following high-risk pregnancies, suggesting a potential prenatal origin for their elevated metabolic risk. This observation aligns with Barker’s hypothesis, which posits that adverse conditions during gestation, such as those seen in complicated pregnancies, may increase susceptibility to chronic disease later in life (39, 40). These results underscore the possible impact of prenatal factors on the development of metabolic health in adolescence.

As noted above, HFC further distinguished the MUO group, with significantly elevated levels quantified by MRS, a reference standard for the noninvasive measurement of liver steatosis (32, 33), and corroborated by raised liver enzymes and ultrasound indicators of hepatic steatosis. Although the cross-sectional design of the present study precludes causal inference, this finding suggests that liver fat play a pivotal role in distinguishing metabolic phenotypes within the context of obesity, surpassing the influence of visceral fat, which did not significantly differ between the two groups of adolescents. These findings are consistent with previous trials in adults which demonstrated that hepatic and not visceral fat is strongly linked to obesity-related metabolic complications (38, 41). Our regression models reinforce the independent significance of HFC, even after adjusting for various covariates, our results consistently demonstrated a substantial association between elevated HFC and metabolically unhealthy obesity, these findings emphasize the potential clinical significance of assessing liver fat as an independent biomarker for early identification and intervention in obese adolescents at risk of metabolic disease. It is worth noting that the consensus-based MHO definition currently does not incorporate hepatic steatosis. Given our findings and those of previous studies (38, 42, 43), there is a growing rationale for considering the inclusion of hepatic steatosis in the definition of MHO. However, since liver fat content is not currently part of standard MHO criteria, further studies are needed to validate its predictive value and determine optimal threshold levels for clinical classification.

The precise underlying mechanisms that link metabolic health and hepatic steatosis remain incompletely clarified, but the liver is recognized as playing a pivotal role in governing both carbohydrate and lipid metabolism through an intricate network of metabolic pathways. Primarily, ectopic accumulation of lipids in the liver is closely associated with metabolic dysfunction, leading to MASLD, which exerts a profound impact on the metabolic profile (44, 45). MASLD involves the accumulation of ceramides and diacylglycerols due to excessive free fatty acid accumulation, triggering insulin resistance through insulin receptor dysfunction and downstream signaling pathways (46). The capability of insulin to inhibit hepatic glucose production is compromised in the presence of MASLD-associated insulin resistance, leading to worsening glycemic control. Simultaneously, the suppression of adipose tissue lipolysis is inhibited, perpetuating a vicious cycle of insulin resistance and heightening the risk of cardiovascular complications (47, 48). In line with this perspective, another mechanistic explanation contributing to the metabolically unfavorable profile in MASLD involves the secretion of inflammatory cells and cytokines, known as hepatokines (45). Among these hepatokines are fetuin A, follistatin, HFREP1, LECT2, PEDF, and ectodysplasin, collectively exacerbating insulin resistance in skeletal muscle and adipose tissue through the activation of the c-Jun N-terminal kinase signaling pathway. This pathway is characterized by direct inhibitory phosphorylation of insulin receptor substrates, resulting in diminished insulin signaling and exacerbation of hyperglycemia (45).

Our dietary analysis revealed significant differences in nutrient intake between the two study groups. Adolescents with MUO had a significantly higher total calorie intake, suggesting overconsumption as a contributing factor to metabolic syndrome among them. Furthermore, their dietary patterns were characterized by elevated protein intake, particularly from animal sources, alongside increased consumption of red meat. The detrimental association of high meat consumption on health is well-documented in adults, contributing to metabolic alterations such as insulin resistance and associated diseases such as T2DM (49, 50), MS (51), cardiovascular disease (52, 53), and colorectal cancer (54, 55). A recent study revealed an association between the intake of total, red, and/or processed meat and the incidence and persistence of MASLD, along with clinically significant fibrosis in adult populations (56). This heightened risk may be attributed to several factors, including the impairment in insulin signaling induced by palmitic acid (16:0), which is the most abundant SFA found in animal-derived foods (57–60). Additionally, the consumption of SFAs has been linked to elevated circulating levels of total lipoprotein and LDL-c (61, 62). While our analysis did not find a significant difference in total SFA intake between groups (*p* = 0.09), there was a significant increase in the consumption of major long-chain SFAs, specifically, palmitic and stearic acid (18:0), in the MUO group. These long-chain SFAs were observed as being particularly detrimental to metabolic health compared to short-medium SFAs in a recent systematic review (63). Moreover, the detrimental effect of red meat products can be related to specific cooking methods, such as advanced glycation end products, heterocyclic amines, heme iron and other byproducts of muscle protein oxidation, adds to these risks (53, 64–66).

Interestingly, we observed significantly higher consumption of arachidonic acid among adolescents with MUO, which positively correlated with their intake of animal protein and red meat (67). A major role of arachidonic acid is that of a substrate for the synthesis of eicosanoids, which include prostaglandins, thromboxanes, and leukotrienes. These are formed by the metabolism of arachidonic acid by cyclooxygenase, lipoxygenase, and cytochrome P450 pathways (68–70). The resulting metabolites have many roles in inflammation, regulation of the immune response, blood clotting, and smooth muscle contraction (68–70). While the functions of arachidonic acid-derived metabolites are well-established in human health outcomes, recent reviews on the impact of arachidonic acid consumption in adults indicated no adverse effects from their increased intake (71). Also, while there was no significant difference in overall ultra-processed food intake between our two study groups, the MUO group showed a notable increase in the consumption of ultra-processed grains, including white bread and rolls, pastries, sugary breakfast cereals, and more. This observation underscores the negative impact of ultra-processed grains, emphasizing their negative contribution to health outcomes. This effect is evident both independently and when combined with a Western diet that is characterized by high consumption of red and processed meats, sugary snacks and drinks, refined grains, convenience foods, and low intake of fruits, vegetables, and whole grains (72, 73). Sodium intake was significantly higher among our adolescents with MUO compared to those with MHO, and nearly double the recommended daily upper limit of 2,300 milligrams per day (74). Sodium is widely recognized for its substantial role in blood pressure and metabolic health dysregulation, even among adolescent populations (75, 76).

Lastly, our evaluation of physical activity and performance revealed unexpectedly insignificant differences between the MUO and MHO groups. Contrary to prevailing beliefs (77, 78), both groups displayed similar levels of physical activity and sedentary behavior with high levels of sedentary behavior and no regular physical exercise. All participants spent an average of approximately 60% of their day in sedentary pursuits, far exceeding the recognized cutoff that leads to increased cardiovascular disease risk in adults (79). These results highlight the need for further investigation, with larger number of participants, in order to assess the relationship between obesity phenotype and physical activity levels. The inclusion of hand grip strength and isometric mid-thigh pull max test in our study can help to establish a normative range for adolescents with obesity and furnish informative benchmarks for future research and clinical evaluations.

One strength of our study is our employment of advanced imaging techniques, including MRI and MRS, which are considered gold standards and references for assessing body fat distribution and HFC, respectively. This approach allowed precise and reliable measurements, thereby enhancing the validity of our results. Moreover, the study population of adolescents with obesity provides a unique perspective in understanding the early markers of unhealthy metabolic obesity and valuable insights for developing interventions to prevent metabolic abnormalities during development. Another strength lies in our examination of a wide range of variables, from prenatal factors, such as birth weight and pregnancy conditions, to current markers, such as blood parameters, physiological measures, and dietary patterns assessed by the FFQ.

Several limitations include the cross-sectional design and relatively small sample size. While efforts were made to adjust for confounding factors, it is possible that our model did not fully account for all contributors to metabolic obesity as demonstrated in this study. Therefore, caution is warranted when interpreting the independent influence of HFC, since unmeasured covariates (such as genetic factors and environmental influences) may have influenced the observed associations. Additionally, there may be information bias in dietary self-reporting due to recall bias. We attempted to bridge this gap by using FFQ, which is a reliable and widely accepted tool for assessing dietary patterns in children.

In conclusion, our findings demonstrate a significant association between hepatic fat content (HFC) and metabolic health status in adolescents with obesity, along with distinct differences in dietary patterns and maternal risk factors between metabolically unhealthy and healthy obese subgroups. Although causality cannot be inferred due to the cross-sectional design of the study, the observed associations suggest that HFC may serve as a meaningful marker of metabolic risk in this population.

While no universally accepted HFC threshold currently exists for guiding pediatric intervention, our results support the clinical utility of the >5.5% cutoff—commonly used to define hepatic steatosis—as a pragmatic reference point. We propose that pediatricians consider this threshold as an indicator for initiating early, individualized lifestyle interventions, including dietary counseling, reduction of salt and red or processed meat intake, and increased physical activity. These recommendations may help translate imaging-based hepatic assessments into preventive strategies for at-risk adolescents.

Longitudinal studies in larger, more diverse populations, using more objective dietary methods such food diaries, are essential to validate these findings and to establish evidence-based guidelines for clinical practice.

## Conclusion

- Distinct subgroup of youth with obesity maintains a metabolically healthy profile, though the factors contributing to this status remain incompletely understood.
- Hepatic fat content, but not adipose fat distribution emerged as health marker distinguishing between metabolically healthy and unhealthy obese adolescents.
- Diet that contains ultra processed grains, animal protein and sodium may exert a notable influence on metabolic health during early stages of life.

## Data Availability

The raw data supporting the conclusions of this article will be made available by the authors, without undue reservation.
